# Explainable AI based cervical cancer prediction using FSAE feature engineering and H2O AutoML

**DOI:** 10.1038/s41598-025-23593-9

**Published:** 2025-11-18

**Authors:** Panneerselvam Karthikeyan, I. Malaserene, E. Deepakraj

**Affiliations:** School of Computer Science Engineering and Information Systems, Vellore Institute of Technology, Vellore, India

**Keywords:** Cervical Cancer, Machine Learning, H2O AutoML, LIME, Predictive Analytics, Deep Learning, Healthcare Applications, Risk Prediction, Model Interpretability, HPV, Evolutionary developmental biology, Applied mathematics, Computational science, Computer science, Information technology, Scientific data, Statistics, Biotechnology, Cancer, Computational biology and bioinformatics, Engineering, Mathematics and computing

## Abstract

Cervical cancer, predominantly caused by Human Papillomavirus (HPV) infection, remains a significant global health burden for women, contributing to elevated morbidity and mortality rates. Early and accurate prediction is critical in improving patient outcomes and optimizing healthcare resource allocation. While machine learning (ML) and deep learning (DL) methods–such as support vector machines, random forests, and convolutional neural networks–have demonstrated promise in disease prediction, model interpretability, computational efficiency, and rely on large, labeled datasets. Additionally, conventional diagnostic methods like piezoresistive, piezoelectric, and optical lever techniques are often cost-prohibitive and complex, limiting widespread use. This study proposes a hybrid ML framework that integrates H2O AutoML with an autoencoder-based feature extraction and Fisher Score-based feature selection. To enhance model transparency and clinical trust, Local Interpretable Model-Agnostic Explanations (LIME) and SHAP (SHapley Additive exPlanations) are employed. The workflow initiates with exploratory data analysis (EDA) and dimensionality reduction using a stacked autoencoder, followed by selection of the top predictive features via Fisher Score. The refined feature set is used to train multiple models via H2O AutoML, with the best-performing deep learning model selected. On the training dataset, the selected model achieved 95.24% accuracy, an AUC of 98.10, and a log loss of 0.1747. Cross-validation confirms the model’s robustness with consistent AUC and log loss values. At the optimal F1 threshold of 0.517, the confusion matrix indicates an error rate of 5.75% for actual negatives and 2.59% for actual positives, leading to an overall error rate of 4.14%. LIME and SHAP are used to interpret predictions at the instance level, providing actionable insights for clinicians. These results demonstrate the effectiveness of combining AutoML with explainable AI and advanced feature engineering to enhance the predictive power and interpretability of cervical cancer risk models, offering a scalable solution for clinical decision support.

## Introduction

**Fig. 1:**
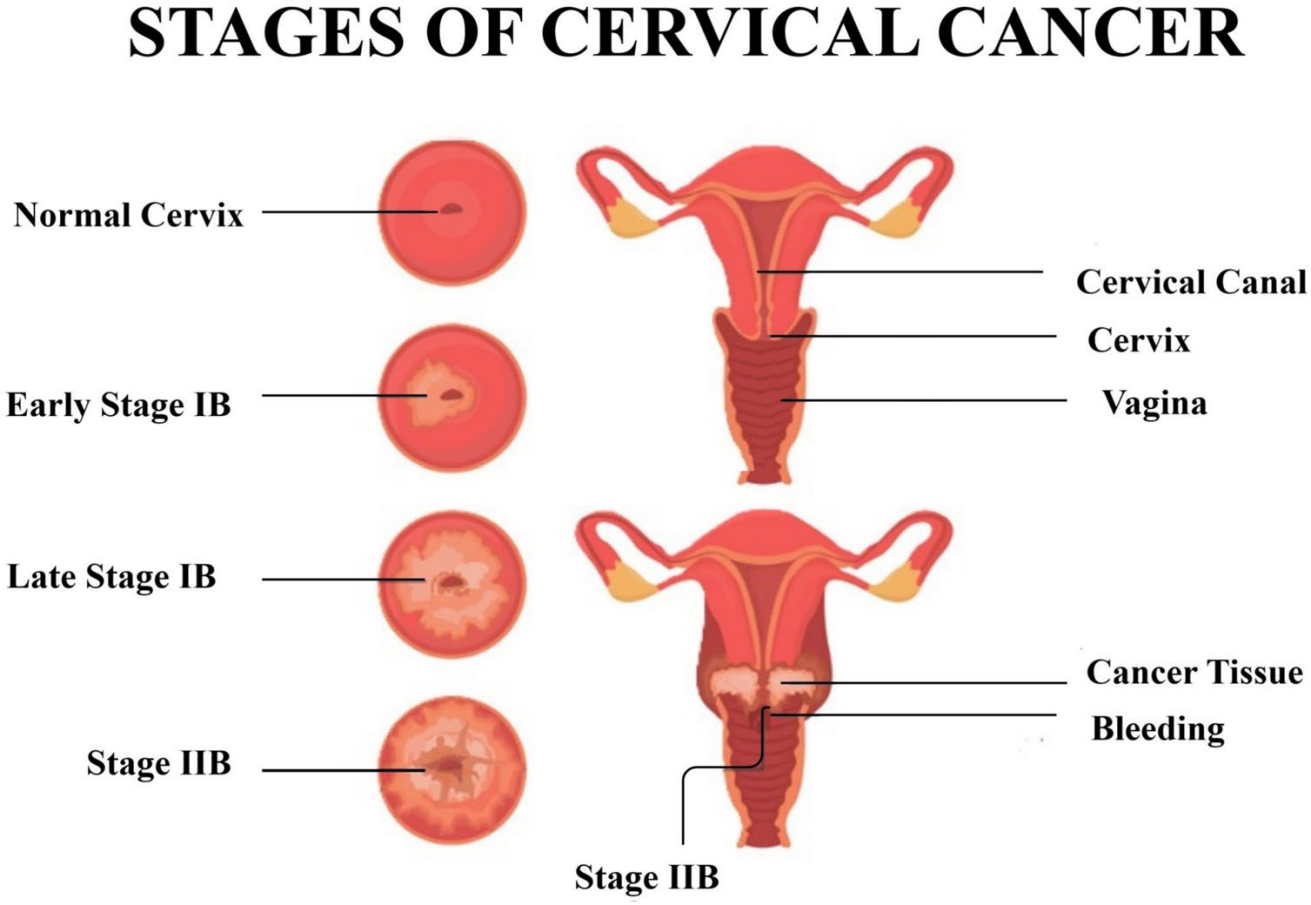
Anatomy of a healthy cervix and cancerous tissue showing early (Stage IB) and advanced stages (Stage IIB).

Cervical cancer remains a major public health problem worldwide, particularly in low- and middle-income countries where screening and vaccination programs may be limited. According to the World Health Organization (WHO), cervical cancer is the fourth most common cancer among women, leading to approximately 311,000 deaths worldwide in 2020^1^. The primary cause of cervical cancer is persistent infection with high-risk types of human papillomavirus (HPV), a sexually transmitted virus. The anatomy of a healthy cervix along with the progression of cervical cancer through various stages such as Early Stage IB, Late Stage IB and Stage IIB has been detailed in Fig. 1.

A healthy cervix has normal tissues with well-defined structures and no signs of abnormalities. In early stage cancer (Stage IB), the disease is confined to the cervix but invades deeper layers of cervical tissue, indicating localized growth. As cancer progresses to advanced stages (Stage IIB), it extends beyond the cervix to the surrounding parametrial tissue, demonstrating significant local invasion without metastasis to distant organs^2^. This gradual transformation underscores the invasive nature of the disease. Despite advances in early detection and prevention strategies, such as Pap smear and HPV vaccination, the incidence of cervical cancer remains alarmingly high in certain regions, which requires the development of effective predictive models to improve patient outcomes^3^. Machine learning (ML) has emerged as a powerful tool for predicting health outcomes and improving clinical decision-making^4^. The ability of ML algorithms to analyze large data sets and uncover complex patterns makes them particularly suitable for oncology, where disease progression factors are multifaceted^5^.

Recent advances in ML, particularly through platforms such as H2O AutoML, allow researchers and healthcare professionals to deploy sophisticated algorithms without requiring extensive programming knowledge. This democratization of AI technology has the potential to revolutionize the way clinicians assess risk and tailor treatment plans for patients, particularly in the context of cervical cancer^6^. In the current landscape of predictive modeling in healthcare, integrating explainable artificial intelligence (XAI) techniques, such as Local Interpretable Model-agnostic Explanations (LIME), is crucial to cultivating trust among clinicians and patients^7^. Although many ML models excel in predictive accuracy, their “black-box” nature can hinder adoption in clinical settings, where interpretability is paramount^8,9^. Using LIME, we achieve the transparency of predictive models, providing insight into how specific characteristics influence decisions, which is critical for informed clinical decision making^10^.

### Importance of predictive modeling for cervical cancer

Predictive modeling offers transformative potential in the early detection and personalized management of cervical cancer. Using demographic data with demographic data from patients, clinical history, lifestyle factors, and screening results, ML models can identify individuals at higher risk with greater precision than traditional methods. This enables stratified screening schedules, improved resource allocation, and reduced unnecessary procedures for low-risk individuals.

High-risk individuals may benefit from timely interventions such as advanced diagnostic imaging or targeted biopsies. In resource-limited regions, predictive models can serve as diagnostic tools to assess prioritizing follow-up care and also support shared decision-making, improving prognosis and care quality. In public health contexts, these models help identify population-level risk trends and disparities, aiding targeted interventions and policy development.

### Challenges in cervical cancer prediction

Despite promise, several challenges hinder the integration of ML into clinical workflows. Data-related issues include small dataset sizes, class imbalance, and lack of demographic diversity. These affect the generalizability of the model and the fairness of the model. Data heterogeneity structured data, unstructured text, and images complicate model integration.

Privacy concerns and regulatory barriers limit data sharing, impeding the creation of large-scale and diverse datasets. Technically, issues such as overfitting, interpretability, and real-world validation remain problematic. Interdisciplinary collaboration is essential to bridge the gap between algorithm development and clinical application, ensuring that models are useful and reliable in practice.

### The role of H2O AutoML and LIME

H2O AutoML streamlines the development of robust ML models by automating pre-processing, model selection, tuning, and evaluation. It allows domain experts to develop high-performing models without extensive programming.

In cervical cancer prediction, H2O AutoML can test various algorithms efficiently to identify the best fit, In terms of accuracy. However is not enough in healthcare. LIME (Local Interpretable Model-agnostic Explanations) complements AutoML by making model decisions transparent. It explains individual predictions by approximating them with interpretable models, enhancing trust and clinical adoption.

LIME identifies characteristics (e.g. HPV status, age.. etc) which influence outcomes, ensuring that the model is understandable and reliable. This is crucial for regulatory compliance, clinician participation, and ethical use of AI in medicine.

### Objectives of the study

This study is centered on four main objectives:

1. **Data understanding and preprocessing:** Conduct a thorough EDA to assess data quality, handle missing values, and prepare the dataset for modeling.
2. **Model development using H2O AutoML:** Use H2O AutoML to build an accurate and efficient predictive model to assess the risk of cervical cancer.
3. **Model interpretability with LIME:** Apply LIME to ensure transparency by identifying influential features for individual predictions.
4. **Clinical Relevance and Integration:** Evaluate the practical value of the model and propose integration strategies into real-world screening protocols, particularly in low-resource settings.

By fulfilling the above objectives, the study aims to provide a practical, interpretable and scalable ML framework to support early diagnosis, risk stratification, and better patient management in cervical cancer care.

### Problem statement and study boundaries

This study addresses the challenge of accurately predicting the result of cervical cancer biopsy outcomes using tabular screening data. Using a data set comprising 36 clinical and demographic characteristics, the objective is to develop predictive models capable of identifying high risk, thus facilitating timely intervention and supporting clinical decision making. The scope of this work is restricted to tabular risk prediction on the publicly available Kaggle dataset. Imaging modalities, multiclass staging of cervical cancer, and prospective clinical trials are not considered. Consequently, the findings should be viewed as a demonstration of methodological feasibility within an experimental research setting, rather than as a fully deployable clinical decision support tool.

## Related work and literature survey

During the past decade, significant strides have been made in applying deep learning techniques to the diagnosis and classification of cervical cancer. Traditional diagnostic techniques such as Pap smear cytology have been enhanced with computer-aided approaches, particularly Convolutional Neural Networks (CNNs), to improve accuracy and reduce human error. Alyafeai and Ghouti^11^ proposed a fully automated deep learning pipeline that uses architectures to handle the inherent complexity of cervical cell images, such as overlapping structures, variations in staining, and morphological similarities between benign and malignant cells. Their approach demonstrated improved segmentation and classification capabilities, which are crucial for early-stage cancer detection.

Habtemariam et al.^12^ extended this work using deep convolutional networks on cervical cell image datasets to classify cancerous and non cancerous samples. Their model achieved high accuracy and sensitivity, confirming CNNs’ viability in real-world diagnostics. However, one persistent challenge is class imbalance, especially when datasets contain a small proportion of malignant cases. Pacal and Klcarslan^13^ addressed this using geometric data augmentation techniques to synthetically balance the data set, improving the generalization of the model and reducing the bias of the majority class.

In parallel, eXplainable Artificial Intelligence (XAI) has emerged as an essential component for clinical trust and regulatory compliance. Hussain et al.^14^ introduced XAI in the diagnosis of Intraepithelial Cervical Neoplasia (ICN), offering heat maps and importance metrics of features for clinical interpretation. Similarly, Di Giammarco et al.^15^ utilized Grad-CAM and SHAP to not only but model predictions that accurate but also interpretable.

Broader developments in AutoML, XAI, and federated learning further inform the direction of AI in healthcare. Waring et al.^16^ provided a comprehensive review of AutoML systems in clinical settings, highlighting their ability to reduce manual tuning. Ooms^17^ demonstrated the usability of AutoML as a self-service tool for clinicians. Reddy et al.^18^ introduced a customized AutoML pipeline for healthcare care, while Rashidi et al.^19^ provided a supervised learning primer for laboratory medicine professionals who integrate AutoML into diagnostics.

Gupta and Seeja^20^ performed a comparative analysis of XAI models (SHAP, LIME, Grad-CAM), emphasizing interpretability in clinical workflows. Tjoa and Guan^21^ provided a foundational survey on XAI, specifically its medical applications. Taniguchi et al.^22^ applied explainable models for ECG-based atrial fibrillation detection, demonstrating real-world feasibility. Meanwhile, Sandhu et al.^23^ explored federated learning for medical imaging, promoting the training of privacy-preserving model in decentralized data sets.

AutoML benchmarking efforts, such as those by Romero et al.^24^ and Chitara et al.^25^, evaluated toolkits such as H2O and Auto-sklearn for disease prediction. Zhuhadar and Lytras^26^ specifically reviewed AutoML in diabetes diagnosis, focusing on sustainability and model reliability. Siriborvornratanakul^27^ examined human-in-the-loop behavior in AutoML-enhanced diagnostic systems.

Together, these works underscore the transformative potential of AutoML and XAI in healthcare while reinforcing the need for interpretability, trust, and clinical integration.

**Table 1:** Summary of recent literature on AutoML, XAI, and federated learning in healthcare.

Study	Methodology	Key Contributions	Ref. No
Alyafeai and Ghouti (2020)	CNN-based segmentation	Fully automated pipeline for cervical image classification	^11^
Habtemariam et al. (2022)	Deep CNN classification	High sensitivity in cervical cancer diagnosis	^12^
Pacal and Kılıcarslan (2023)	Data augmentation	Tackled class imbalance in cervical datasets	^13^
Hussain et al. (2024)	XAI for CIN	Clinical interpretability via heatmaps and SHAP values	^14^
Di Giammarco et al. (2023)	Grad-CAM + SHAP	Enhancing model trustworthiness	^15^
Waring et al. (2020)	AutoML Review	Outlines AutoML challenges and potential in clinical settings	^16^
Gupta and Seeja (2024)	XAI comparison	Evaluates SHAP, LIME, Grad-CAM for medical diagnostics	^20^
Tjoa and Guan (2020)	XAI survey	Overview of interpretability in medical AI models	^21^
Sandhu et al. (2023)	Federated Learning	Preserving patient data privacy across sites	^23^
Ooms (2019)	AutoML thesis	Self-service AutoML in healthcare environments	^17^
Reddy et al. (2025)	Custom AutoML pipeline	Contextualized AutoML in cognitive healthcare apps	^18^
Rashidi et al. (2021)	AutoML in lab medicine	Educational primer for ML in diagnostics	^19^
Romero et al. (2022)	AutoML benchmarking	Compared frameworks for medical claims analysis	^24^
Zhuhadar and Lytras (2023)	AutoML for diabetes	Review of AutoML accuracy and sustainability	^26^
Siriborvornratanakul (2022)	Human-AI interaction	Behavioral factors in AutoML-driven diagnostics	^27^

As summarized in Table 1, various studies have applied AutoML and XAI techniques in healthcare, specifically in areas such as cervical cancer diagnosis, federated learning, and disease prediction.

## Research gap

Despite promising advances in automated cervical cancer diagnosis, several key research gaps persist that limit the clinical applicability and robustness of current AI-driven solutions.

Firstly, most studies focus on binary classification (normal vs. abnormal or benign vs. malignant), neglecting the more nuanced task of multiclass classification. Accurate differentiation between various stages of cervical intraepithelial neoplasia (CIN I, II, III) or subtypes of cervical cancer (such as squamous cell carcinoma and adenocarcinoma) remains a significant challenge. A lack of well-annotated stage-specific data sets contributes to this limitation and hampers the development of comprehensive models capable of real-world diagnosis.

Secondly, while explainability tools have been introduced, many models still act as ’black boxes,’ offering limited insight into the contributions of features, especially in complex classification tasks. Deeper integration of interpretable AI frameworks that provide both global (data set level) and local (individual prediction level) explanations is essential to improve trust, especially in high-stakes medical environments. Another critical gap lies in the robustness of these models in varying image quality and populations. Many data sets are collected in controlled environments and may not reflect real-world diversity in terms of ethnicity, age, or imaging equipment. Consequently, models trained on such datasets often perform poorly when deployed in different clinical settings.

Furthermore, current models rarely address cost-sensitive learning, which is crucial in healthcare contexts where the cost of false negatives (i.e. missed cancer diagnoses) are significantly higher than false positives. Incorporating asymmetric cost functions can help balance prediction sensitivity and specificity based on clinical risk. Lastly, federated learning, where models are trained across decentralized data sources without sharing sensitive patient information, remains underexplored in cervical cancer research. This technique has immense potential to aggregate insights from diverse healthcare institutions while preserving patient privacy, a key requirement for large-scale adoption of AI in healthcare.

Addressing these research gaps is critical for the development of robust, generalized, and clinically reliable predictive models that can be effectively integrated into real-world cervical cancer screening and diagnostic systems.

## Background study

### Cervical cancer

Cervical cancer is a prevalent form of cancer that affects women worldwide, primarily caused by persistent infection with high-risk human papillomavirus (HPV). Early stage cervical cancer often presents limited symptoms, making timely diagnosis challenging. Common detection methods include Pap smears, HPV tests, and colposcopy, which despite their effectiveness, face limitations in sensitivity, cost, and accessibility, particularly in low-resource settings. Consequently, there is growing interest in the development of automated prediction tools that use machine learning to improve the accuracy, accessibility, and interpretability of cervical cancer screening.

### Automated machine learning (AutoML) and explainable AI (LIME/SHAP)

Automated Machine Learning (AutoML) platforms, such as H2O AutoML, streamline the model selection and optimization process, enabling non-experts to develop robust predictive models with minimal manual effort. H2O AutoML automates tasks such as hyperparameter tuning and algorithm selection to maximize performance. In healthcare, the interpretability of the model is crucial for adoption. Local Interpretable Model-Agnostic Explanations (LIME) offers a framework to explain predictions at the individual level, while SHapley Additive Explanations (SHAP) provides a theoretically grounded approach for global and local feature importance. Together, LIME and SHAP offer complementary perspectives for enhancing transparency in clinical decision-making.

### Feature engineering with Autoencoder and PCA

To enhance feature representation and reduce dimensionality, we applied an autoencoder-based feature extraction pipeline. The autoencoder compresses the high-dimensional input features into a lower-dimensional latent space, capturing essential patterns and structure within the data:

1 $$\begin{aligned} z = f_{\text {enc}}(x), \quad \hat{x} = f_{\text {dec}}(z) \end{aligned}$$

where $x$ is the input, $z$ is the encoded latent representation, and $\hat{x}$ is the reconstructed output. The encoder-decoder network is trained to minimize the reconstruction loss:

2 $$\begin{aligned} \mathcal {L}_{\text {AE}} = \frac{1}{N} \sum _{i=1}^{N} \Vert x_i - \hat{x}_i \Vert ^2 \end{aligned}$$

Subsequently, Principal Component Analysis (PCA) was employed to visualize the latent feature space and support data-driven exploratory analysis.

### Feature selection using fisher score

After autoencoder transformation, feature selection was performed using Fisher Score, a supervised filter method that ranks features based on class separation. The Fisher score for the feature $i$ is calculated as:

3 $$\begin{aligned} F_i = \frac{(\mu _i^+ - \mu _i)^2 + (\mu _i^- - \mu _i)^2}{\sigma _i^{+2} + \sigma _i^{-2}} \end{aligned}$$

where $\mu _i^+$ and $\mu _i^-$ are the means of the characteristic $i$ for the positive and negative classes, respectively, and $\sigma _i^{+2}, \sigma _i^{-2}$ are the corresponding variances. This method helps retain features that are most informative for distinguishing cancerous from noncancerous cases.

### H2O AutoML: model pipeline

H2O AutoML automates the training, selection, and optimization of machine learning models. It supports a variety of algorithms including Generalized Linear Models (GLM), Gradient Boosting Machines (GBM), Random Forests (RF), Deep Learning models, and Stacked Ensembles. The AutoML process seeks to optimize a loss function (e.g., log loss) or maximize a performance metric (e.g., AUC):

4 $$\begin{aligned} h^*= \arg \min _{h \in \mathcal {H}} \frac{1}{|\mathcal {D}_{\text {val}}|} \sum _{(x,y)\in \mathcal {D}_{\text {val}}} \ell (h(x), y), \end{aligned}$$

where $\ell$ is the binary cross-entropy loss:

5 $$\begin{aligned} \ell (\hat{p}, y) = - \big ( y \log \hat{p} + (1-y) \log (1-\hat{p}) \big ). \end{aligned}$$

#### Data splitting

Let the data set be denoted as follows:

- $$D$$: Entire dataset
- $$D_{\text {train}}$$: Training set
- $$D_{\text {val}}$$: Validation set
- $$D_{\text {test}}$$: Test set

These subsets satisfy:

6 $$\begin{aligned} D = D_{\text {train}} \cup D_{\text {val}} \cup D_{\text {test}} \end{aligned}$$

#### Algorithms used: summary table

To ensure a comprehensive evaluation of predictive performance, multiple machine learning algorithms were employed using the H2O AutoML framework. These included Generalized Linear Models (GLM), Gradient Boosting Machines (GBM), Random Forests, Deep Learning networks, and Stacked Ensembles. Each of these algorithms offers unique strengths; GLM provides interpretability and simplicity, GBM captures complex non-linear relationships, Random Forests enhance robustness through ensemble learning, while Deep Learning models are adept at handling high-dimensional data. The Stacked Ensemble model integrates predictions from multiple base learners, often resulting in superior accuracy. A detailed summary of the equations and the respective roles of each algorithm is provided in Table 2, which serves as a quick reference for the mathematical foundations and core functions of these models.

**Table 2:** Summary of machine learning algorithms used in H2O AutoML.

Algorithm	Equation	Purpose/Utility
GLM	$P(y=1|X) = \frac{1}{1 + e^{-(\beta _0 + \sum _{i=1}^{n} \beta _i x_i)}}$	Models linear relationships; interpretable for binary classification
GBM	$\hat{y}^{(t)} = \hat{y}^{(t-1)} + \eta \cdot g_t(X)$	Boosts weak learners; handles non-linearities and interactions
Random Forest	$\hat{y} = \frac{1}{m} \sum _{i=1}^{m} \hat{y}^i$	Ensemble of decision trees; reduces overfitting and increases accuracy
Deep Learning	$a^{[l]} = g(W^{[l]} a^{[l-1]} + b^{[l]})$	Learns complex patterns; effective for high-dimensional data
Stacked Ensemble	$\hat{y} = f_{\text {meta}}(f_1(X), f_2(X), \ldots , f_k(X))$	Combines strengths of multiple models for improved performance

### Advanced machine learning approaches

Recent advances in machine learning have highlighted the role of attention mechanisms and transformer-based models in handling, complex high-dimensional data. The transformer architecture, originally proposed for natural language processing, has been extended to tabular data domains through models such as TabTransformer and FT-Transformer. The core operation of the transformer is the self-attention mechanism, which models pairwise interactions between features. Given an input matrix $X \in \mathbb {R}^{n \times d}$, the scaled dot-product attention is defined as:

7 $$\begin{aligned} \textrm{Attention}(Q, K, V) = \textrm{softmax}\left( \frac{QK^{\top }}{\sqrt{d_k}}\right) V, \end{aligned}$$

where $Q = XW_Q, K = XW_K, V = XW_V$, and $W_Q, W_K, W_V$ are learnable projection matrices. Such mechanisms allow the model to focus on informative subsets of the input and capture long-range dependencies between features. For tabular biomedical data, transformers have shown potential to automatically learn complex feature interactions.

### Why AutoML with explainable AI was chosen

Despite the promise of transformer-based methods, they present several limitations for the current problem setting. First, the cervical cancer data set used in this study is relatively small, with only 36 input features, which reduces the benefits of deep transformer architectures that typically require large-scale data to generalize effectively. Second, transformer models are computationally intensive and involve numerous hyperparameters, which complicates reproducibility and deployment in resource-constrained clinical environments.

To address these challenges, we adopt an AutoML framework combined with explainable AI (XAI) techniques. AutoML (H2O AutoML in this work) automates the model selection and hyperparameter tuning process, ensuring strong baseline performance across multiple algorithms such as Gradient Boosting Machines, XGBoost, Random Forests, and Deep Learning. This automation reduces the bias introduced by manual tuning and improves the reproductibility of the results. Moreover, AutoML naturally integrates ensemble methods, which are well suited for structured tabular data with moderate sample sizes.

Crucially, the inclusion of XAI methods such as LIME and SHAP provides model interpretability, which is essential in medical applications. These methods enable clinicians to understand both the importance of global features and local prediction explanations, ensuring trust and transparency in the decision support system. Thus, compared to transformer-based models, AutoML combined with XAI offers a more practical, interpretable, and reproducible pipeline tailored to the needs of real-world clinical deployment.

The novelty lies in the integration of the stacked autoencoder, Fisher Score, SMOTE, AutoML, and XAI into one end-to-end pipeline. Each module tackles a different barrier (dimensionality, imbalance, interpretability, deployment). Importantly, SHAP/LIME explanations align with known risk factors, ensuring clinical trust, and the pipeline is deployment-ready (Flask, H2O Wave, low-latency inference). The contribution is therefore the synergy and real-world readiness, not any single routine step.

## Proposed methodology

### System model

**Fig. 2:**
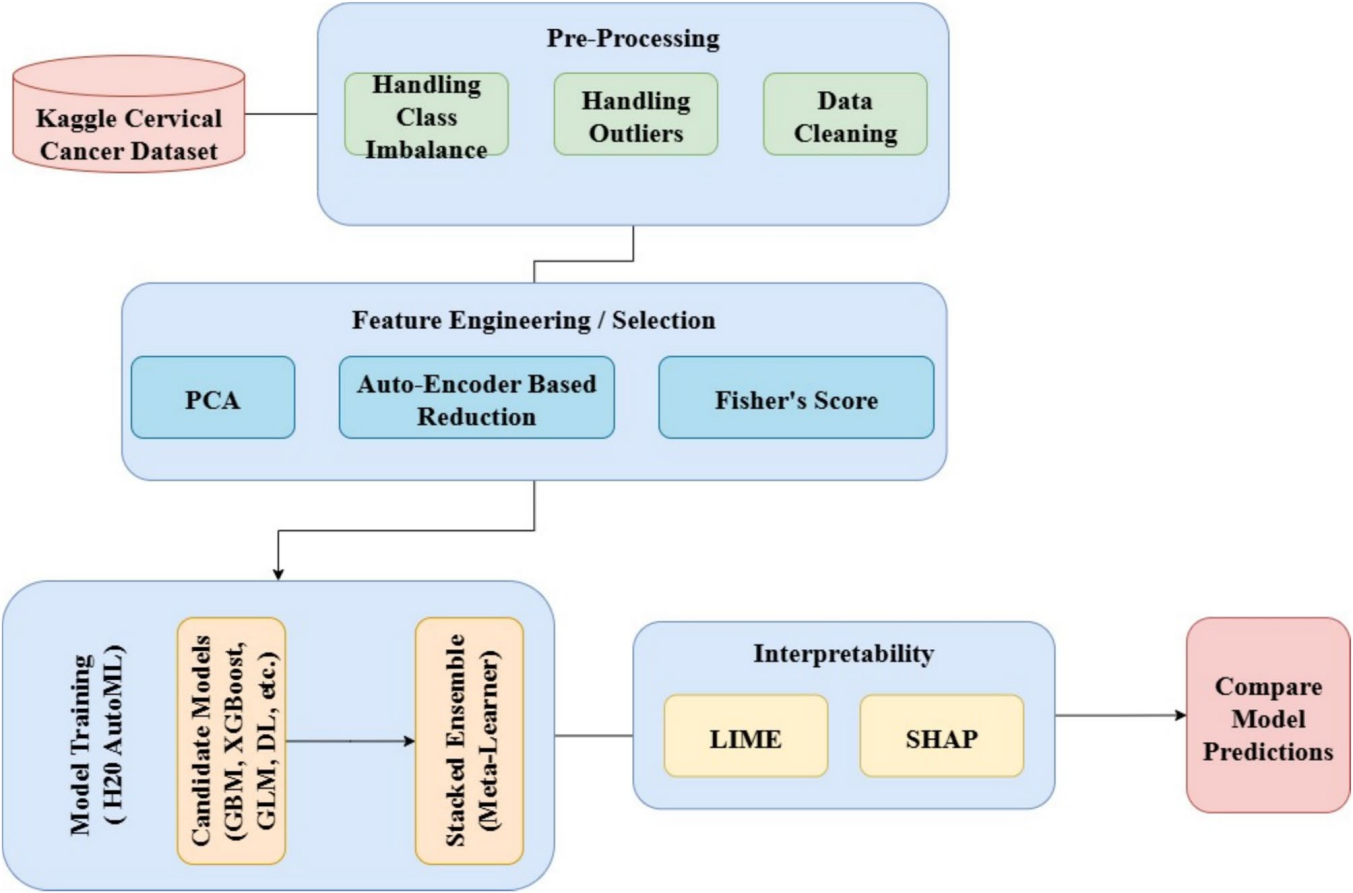
System model of the proposed cervical cancer prediction framework

The proposed system model, shown in Fig. 2, formalizes the end-to-end pipeline for the prediction of cervical cancer. It illustrates how raw screening data are transformed into interpretable outcomes through modular layers ^28^:

- **Input:** Kaggle dataset with 36 demographic and clinical characteristics.
- **Pre-processing:** Data cleaning, outlier handling, normalization, and class balancing using SMOTE.
- **Feature engineering:** Fisher score for feature selection and autoencoder-based dimensionality reduction, with PCA for visualization.
- **Learning:** H2O AutoML trains multiple models (GLM, GBM, RF, XGBoost, DL) and selects the best through stacked ensembles.
- **Interpretability:** SHAP and LIME provide global and local explanations of predictions.
- **Output:** Final predictions with evaluation metrics (accuracy, precision, recall, F1 score, AUC).

This layered design ensures clarity, reproducibility, and clinical relevance, aligning with established system modeling methodologies ^28^.

#### Mathematical formulation of core components

The pipeline can be expressed more formally as follows:

- **Data splitting:** The dataset $\mathcal {D}$ is divided into training, validation, and test sets such that $\mathcal {D} = \mathcal {D}_{train} \cup \mathcal {D}_{val} \cup \mathcal {D}_{test}$ with no overlap.
- **SMOTE oversampling:** For a minority class sample *x* and one of its *k* nearest neighbors $x_{nn}$, a synthetic sample is generated as:
  8 $$\begin{aligned} \tilde{x} = x + \lambda (x_{nn} - x), \quad \lambda \sim U(0,1). \end{aligned}$$
- **Autoencoder:** Each input *x* is encoded into a latent vector and reconstructed as:
  9 $$\begin{aligned} z = f_{enc}(x), \quad \hat{x} = f_{dec}(z), \end{aligned}$$
  with reconstruction loss:
  10 $$\begin{aligned} \mathcal {L}_{AE} = \frac{1}{N}\sum _{i=1}^N \Vert x_i - \hat{x}_i \Vert ^2. \end{aligned}$$
- **Fisher score:** For feature *j*, the score is computed as:
  11 $$\begin{aligned} F_j = \frac{(\mu _j^+ - \mu _j)^2 + (\mu _j^- - \mu _j)^2}{\sigma _{j}^{+2} + \sigma _{j}^{-2}}, \end{aligned}$$
  where $\mu ^+$ and $\mu ^-$ are class means, and $\sigma ^+$, $\sigma ^-$ are class variances.
- **AutoML model selection:** Among candidate models, the optimal model $h^*$ is selected by minimizing validation log-loss:
  12 $$\begin{aligned} h^* = \arg \min _{h \in \mathcal {H}} \sum _{(x,y)\in \mathcal {D}_{val}} \ell (h(x),y). \end{aligned}$$
- **Explainability:** LIME fits a local surrogate model around an instance, while SHAP assigns additive feature contributions such that:
  13 $$\begin{aligned} f(x) = \phi _0 + \sum _j \phi _j. \end{aligned}$$

### Data collection and pre-processing

The cervical cancer data set was sourced from Kaggle^29^, which contains 36 demographic, behavioral, medical, and diagnostic features. The target variable, **biopsy**, indicates the presence (1) or absence (0) of cervical cancer.

Preprocessing was consolidated into a structured pipeline:

- **Missing values:** Converted “?” entries to NaN, followed by mean (continuous) or mode (categorical) imputation.
- **Outlier handling:** Adjusted extreme values using thresholds based on median and median absolute deviation.
- **Feature scaling:** Standardized to zero mean and unit variance.
- **Class balancing:** Applied SMOTE with $k=5$ nearest neighbors to oversample minority cases until an approximate class balance was achieved.

This consolidated summary avoids repetition of preprocessing details in other subsections.

### Exploratory data analysis (EDA)

A comprehensive EDA was conducted to uncover data patterns and distributions, providing critical insights into feature behavior and guiding downstream modeling decisions.

**Fig. 3:**
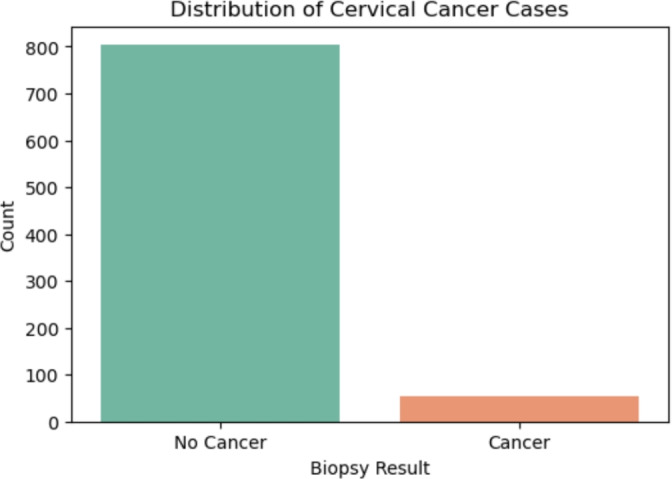
Overall dataset distribution.

**Fig. 4:**
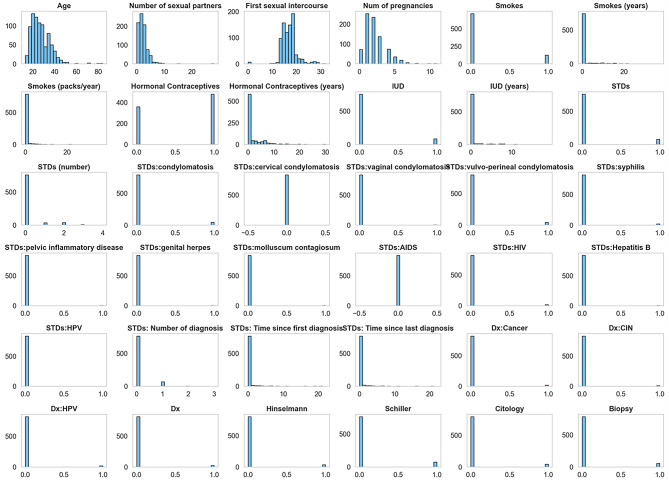
Feature-wise distribution histograms.

**Fig. 5:**
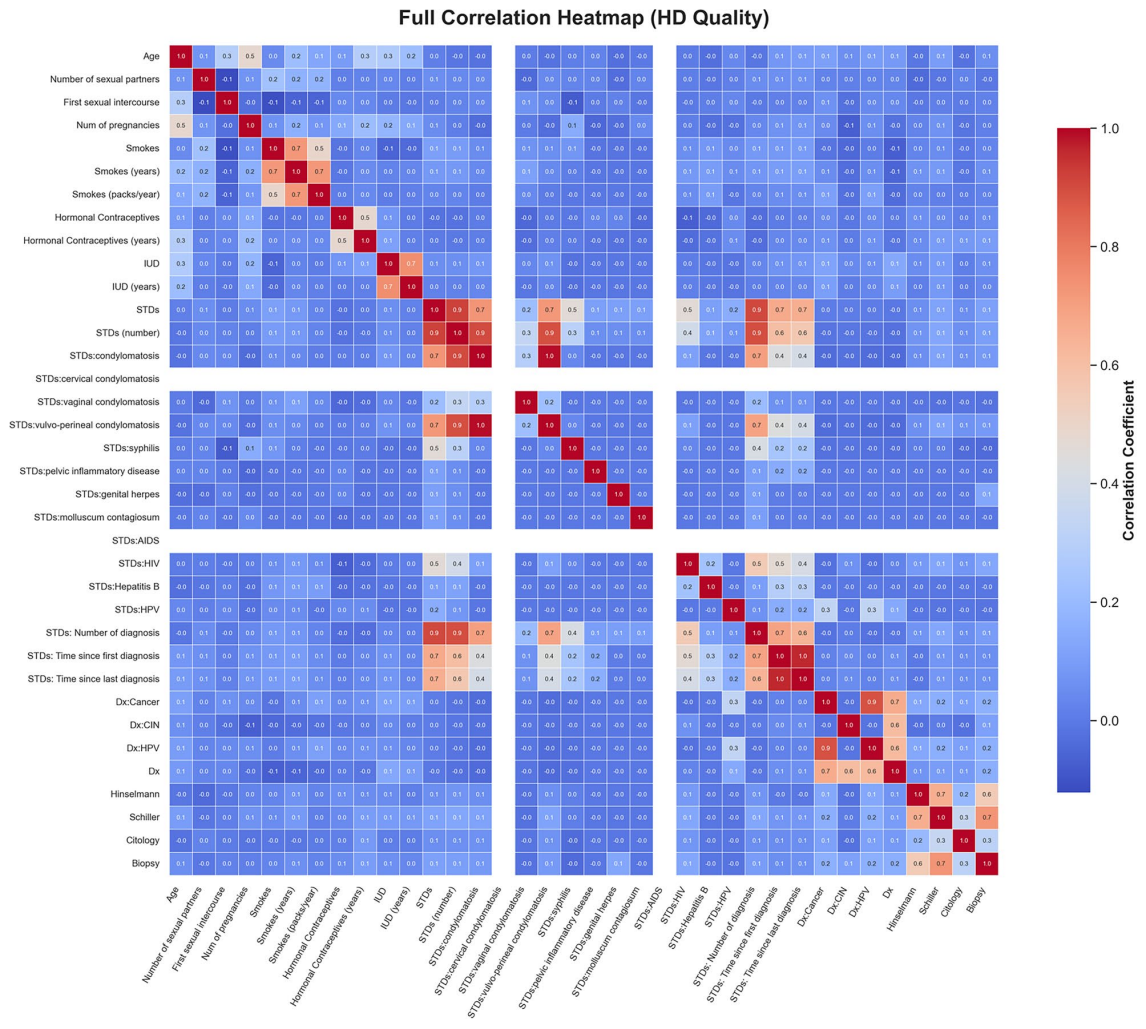
Feature correlation heatmap.

**Fig. 6:**
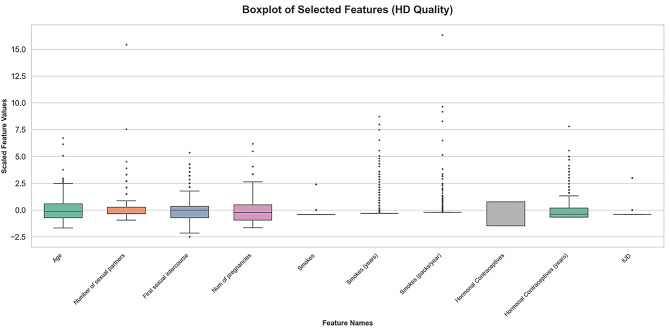
Boxplots of key scaled features.

**Fig. 7:**
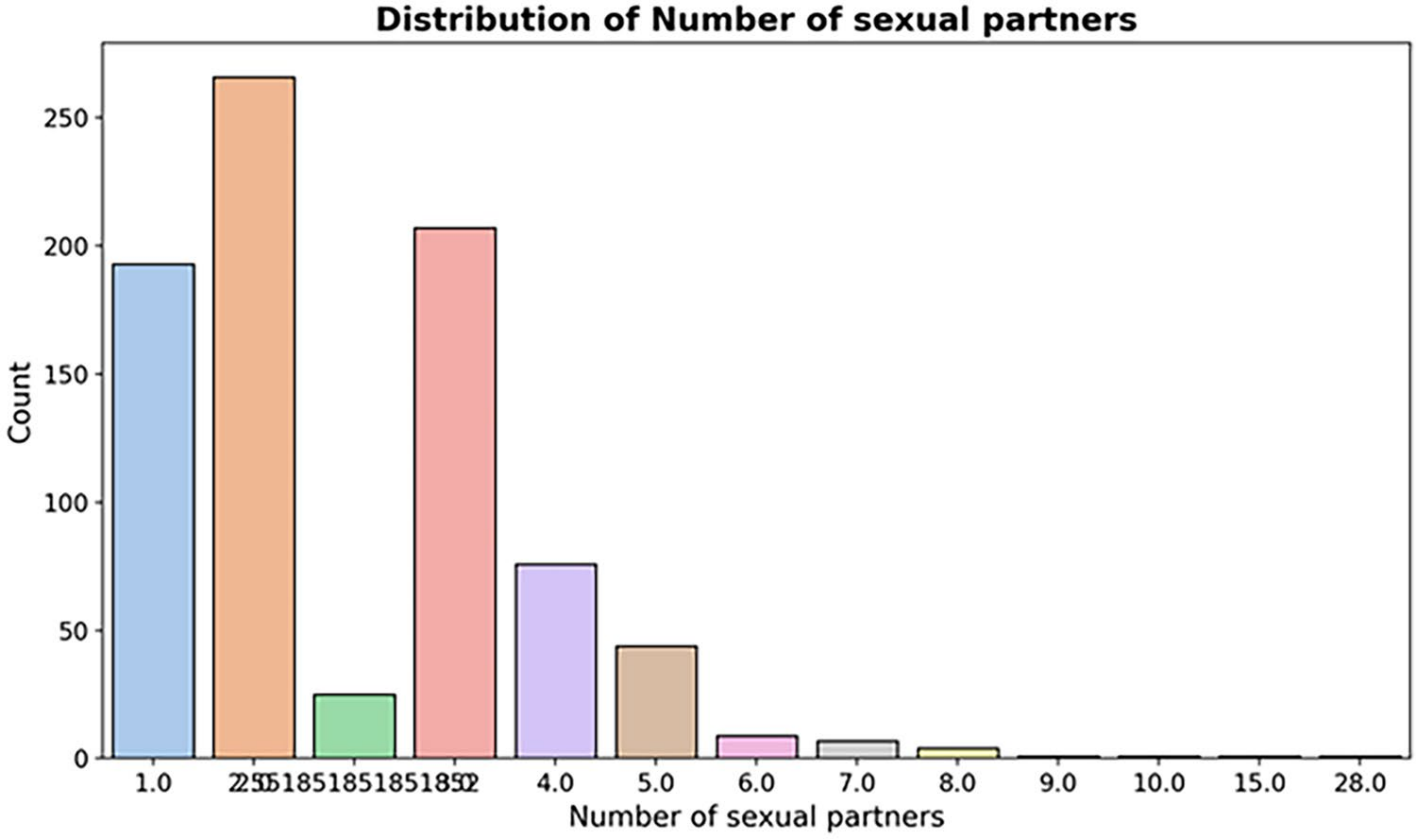
Distribution of number of sexual partners.

- **Dataset distribution:** An overview of the data set value distribution helped to understand the overall data landscape (Fig  3).

- **Feature distributions:** Histograms of individual features (Fig 4) highlighted skewness, kurtosis, and modality, supporting transformation decisions.

- **Feature correlation heatmap:** Pearson correlation coefficients were plotted to identify linear dependencies between features (see Fig  5). This heatmap revealed both highly correlated predictors and those independent of others, essential for dimensionality reduction and multicollinearity checks.

- **Boxplots of key predictors:** Boxplots for the first 10 scaled features illustrated the presence and variance (Fig 6), key for robust preprocessing.

- **Countplots of categorical variables:**
  - *Number of sexual partners* (Fig 7)

- *Smokes* (Fig 8)

**Fig. 8:**
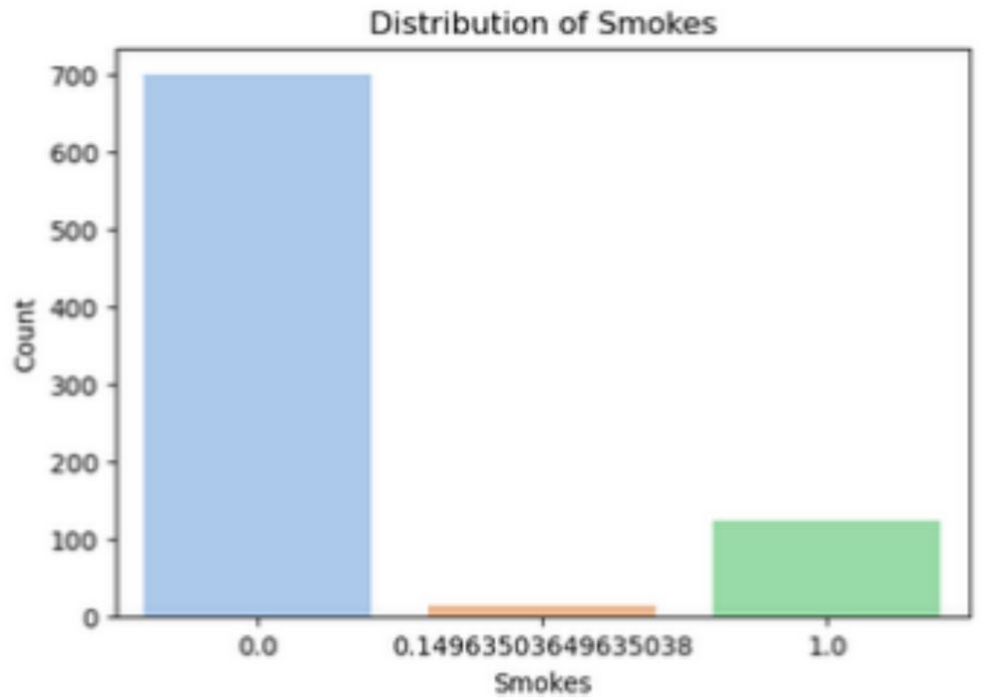
Distribution of smokes.

- *STDs* (Fig 9)

**Fig. 9:**
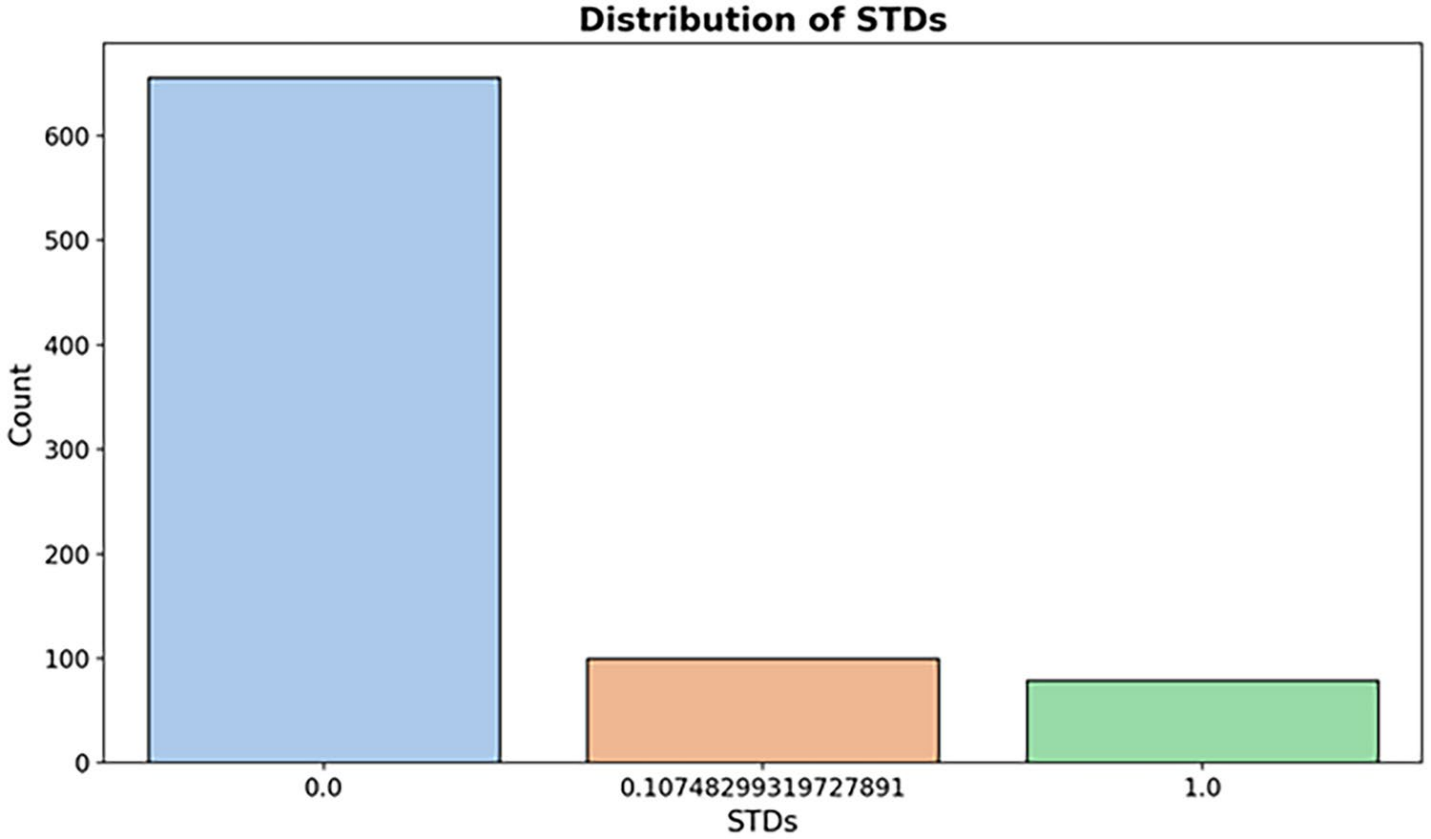
Distribution of STDs.

**Fig. 10:**
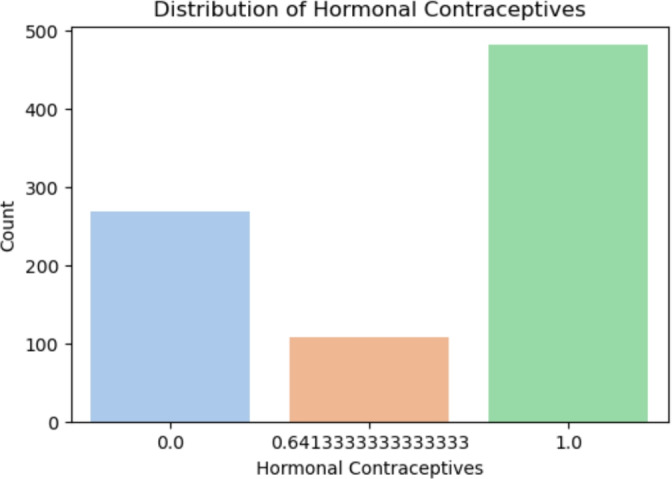
Distribution of hormonal contraceptives.

- *Hormonal Contraceptives* (Fig 10)

- **Target variable distribution:** A countplot of the biopsy outcome revealed a significant class imbalance, which influenced the subsequent use of SMOTE and resampling strategies.

- **Target variable distribution:** A countplot of the biopsy outcome revealed a significant class imbalance, which influence the subsequent use of SMOTE and resampling strategies.

### Feature extraction and selection

To reduce dimensionality and extract robust features, a stacked autoencoder was trained on the standardized data. Encoded representations from the bottleneck layer captured latent features. Fisher Score was then applied to rank and select the most informative features based on class-separation ability.

### Experimental setup

All experiments were carried out on a local workstation with a 12th Gen Intel(R) Core(TM) i5-1240P processor at 1.70 GHz, 16 GB RAM, and a 64-bit Windows 10 operating system. Implementation was done in Python 3.10 with H2O AutoML v3.40, pandas, scikit-learn, and matplotlib.

#### Autoencoder configuration

For feature extraction, an autoencoder was implemented with the architecture and training parameters summarized in Table 3.

**Table 3:** Autoencoder configuration and training parameters.

Parameter	Configuration
Input dimension	36 features
Encoder layers	Dense(16, ReLU) $\rightarrow$ Dense(8, ReLU)
Decoder layers	Dense(16, ReLU) $\rightarrow$ Dense(36, Sigmoid)
Optimizer	Adam (default learning rate = 0.001)
Loss function	Mean Squared Error (MSE)
Batch size	16
Epochs	50
Validation split	20%
Training strategy	Data shuffled at each epoch

#### AutoML configuration

For H2O AutoML, the runtime was restricted to 15 minutes, during which up to 50 candidate models were trained and evaluated. Candidate algorithms included GLM, GBM, Random Forest, XGBoost, Deep Learning, and Stacked Ensembles.

#### Evaluation protocol

To ensure a robust evaluation, repeated 5-fold stratified cross-validation was used. The results are reported as mean ± standard deviation in folds for AUC, f1 score, recall, precision, and log loss.

### Model training using H2O AutoML

The preprocessed data set was transformed into an H2O frame and split into training and test sets. H2O AutoML trained candidate models (GLM, GBM, RF, XGBoost, DL, Stacked Ensembles) within the runtime limit, selecting the best-performing model based on validation AUC and F1 score.

### Evaluation and performance metrics

Performance was assessed using accuracy, f1 score, precision, recall, log loss, matthews correlation coefficient (MCC), and Cohen’s kappa. Visualization included ROC curves, Precision Recall curves, and confusion matrix heat maps.

### Model interpretability with XAI

To ensure transparency and clinical trust, eXplainable AI techniques were employed:

- **LIME:** Generated simplified surrogate models for individual predictions.
- **SHAP:** Quantified contribution of features globally and locally, visualized through force and summary graphs.

### Model insights and feature importance

Understanding model insights is critical to building trust in automated clinical systems. In this work, the importance of the variable extracted from the leader model was analyzed, using the varimp() function, which ranked features according to their contribution to predictive precision. This analysis consistently highlighted demographic and behavioral attributes such as smoking history, contraception use, and history of sexually transmitted diseases (STD), all of which are already established in the medical literature as risk factors for cervical cancer. The convergence between statistical importance and clinical knowledge improves the credibility of the model and provides reassurance to domain experts.

To go beyond global rankings, SHAP analysis was applied to quantify both the direction and magnitude of individual feature effects. This approach revealed, for example, that a longer duration of smoking significantly increased risk predictions, while certain protective behaviors reduced them, which aligns well with epidemiological findings. In contrast, LIME was used to provide local explanations on a case-by-case basis, offering transparency for individual patient predictions. This interpretability is crucial when implementing models in real-world healthcare, as clinicians require both population-level understanding and patient-specific justifications. A correlation and multicollinearity analysis was performed to identify redundant predictors among the top ranked characteristics. This step ensured that the model did not overestimate correlated variables such as overlapping measures of sexual history or contraceptive use, thus preserving interpretive clarity. Together, these complementary interpretability strategies, variable importance, SHAP, LIME, and correlation analysis present a comprehensive view of how the model makes its decisions. Importantly, they also confirm that the model is not only predictive, but also clinically interpretable, making it suitable for integration into decision support systems. These insights strengthen confidence in the model’s outputs and demonstrate alignment with established medical understanding, which is a prerequisite for responsible deployment in sensitive domains Such as Cancer.

### Deployment considerations

Translating predictive models into clinical practice requires careful attention to deployment considerations. Our system was deliberately designed to be lightweight, interpretable, and adaptable for real-world use. The trained model can be exposed through Flask-based REST APIs, which allow seamless integration with electronic health record systems and existing clinical software. In parallel, H2O Wave dashboards provide an intuitive user interface where clinicians can visualize predictions, monitor patient risk scores, and access interpretability reports.This dual deployment approach balances technical scalability with clinical usability.

Interpretability was further embedded into the deployment through the integration of SHAP and LIME explanations directly within the dashboard. This ensures that every prediction is accompanied by a rationale, helping clinicians understand not only the risk estimate, but also the underlying factors driving it. This transparency is key to fostering adoption and reducing resistance among healthcare providers. To maintain fairness and accuracy over time, the pipeline also supports periodic retraining of newly collected data. This continuous learning mechanism prevents performance degradation due to changes in population characteristics or data drift, which are common challenges in medical AI applications. Low-latency inference was prioritized to ensure that predictions can be generated in near-real time, making the tool practical for clinical workflows. The modular design of the system allows individual components–such as the feature engineering pipeline, AutoML module, or interpretability layer–to be updated or replaced without disrupting the entire architecture. Scalability was also considered, with containerized deployment (for example, Docker) enabling portability across different hospital IT environments. By addressing trust, interpretability, latency, and adaptability in deployment, this system moves beyond proof-of-concept experiments and approaches a deployable, clinically relevant decision-support tool. The pipeline is not only technically robust, but also engineered with practical constraints in mind, bridging the gap between academic research and real-world clinical adoption.

## Results

### Explainable AI (XAI) interpretability results

#### LIME-based local interpretations

To enhance the transparency of the model and to facilitate its clinical adoption, Local Interpretable Model-Agnostic Explanations (LIME) were employed. LIME provides patient-specific information by highlighting the most influential features that contribute to the model’s predictions. In the context of cervical cancer prediction, LIME allowed a case-by-case interpretation of the model logic, providing greater confidence in its output.

For example, in one high-risk case, **STI history** emerged as a dominant positive contributor, significantly increasing the prediction of risk. In contrast, **contraceptive use** had a moderate mitigating effect, slightly lowering the risk. Another instance showed **age** and **smoking duration** as key risk-enhancing factors, which align with the established clinical literature linking these features with cervical cancer.

**Fig. 11:**
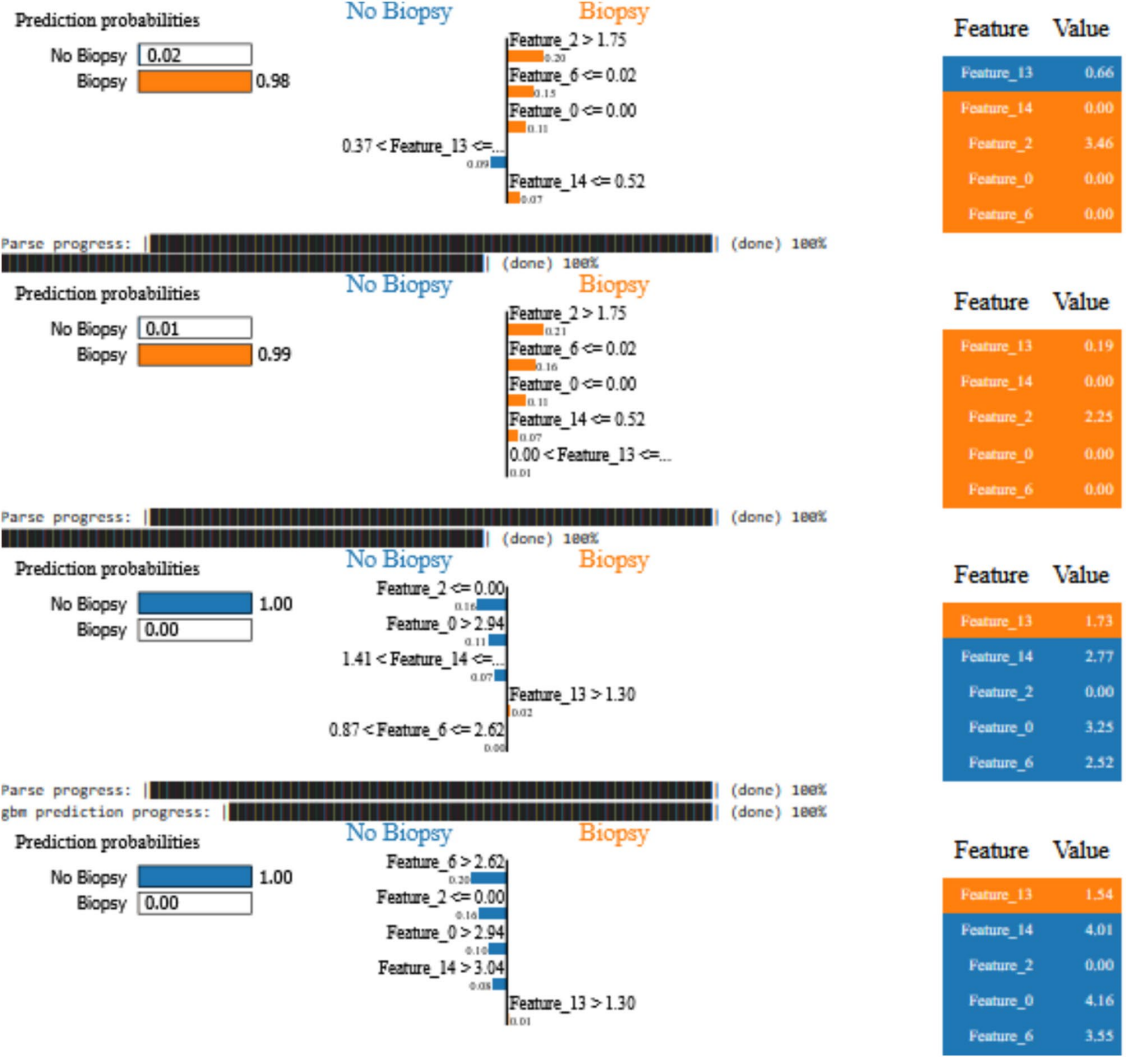
LIME explanation for a cervical cancer prediction instance, demonstrating local feature contributions.

The LIME-based explanation for a specific case is shown in Fig. 11, illustrating the contributions of local features that explain the prediction of the model.

#### SHAP summary for global interpretability

**Fig. 12:**
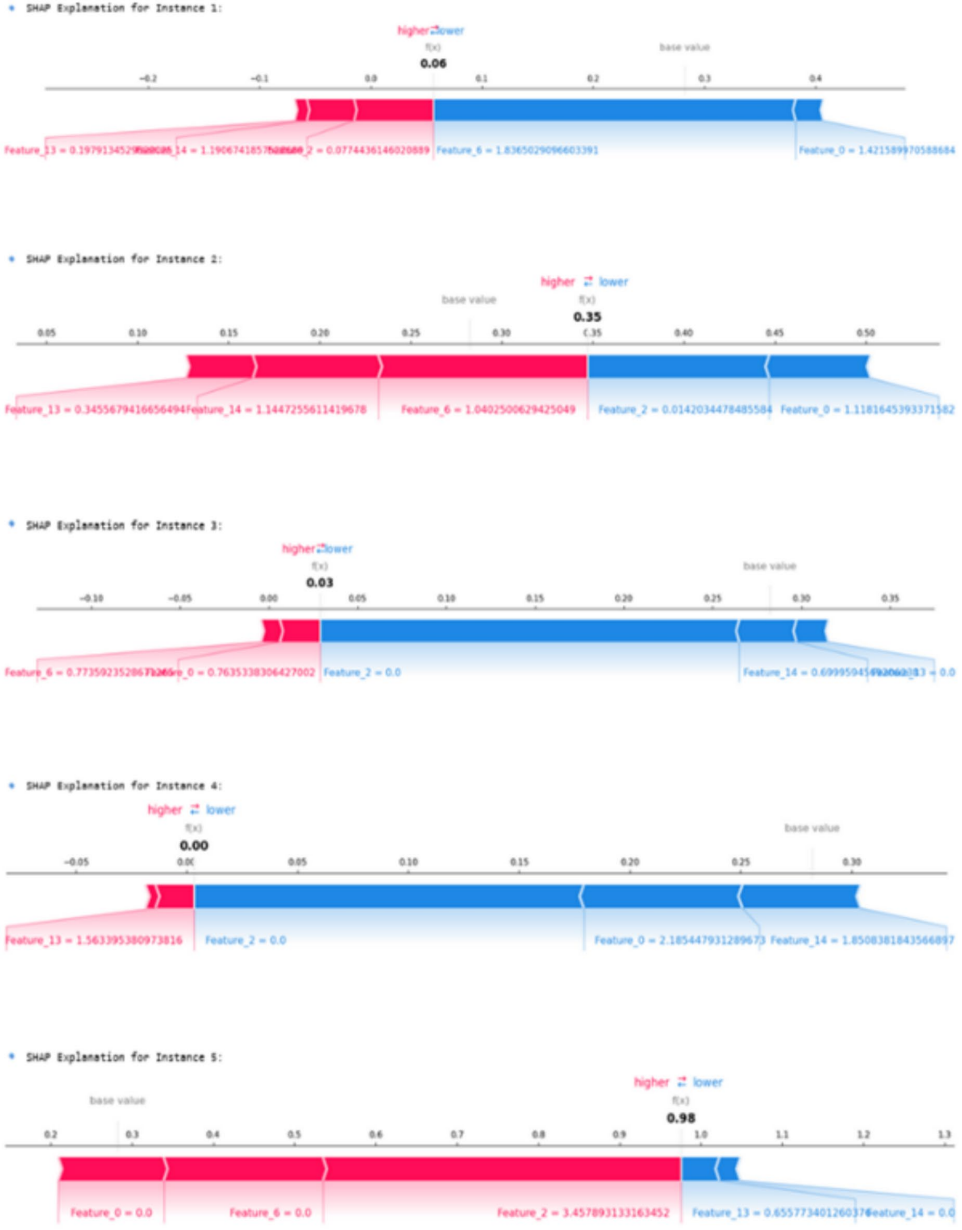
SHAP summary plot highlighting global feature importance for cervical cancer risk prediction.

SHAP (SHapley Additive exPlanations) was used to complement LIME by offering a global interpretability perspective. SHAP provides a broader view of how each feature influences the model output across the entire dataset. The SHAP summary graph, shown in Fig 12, reveals the overall contribution of each characteristic in predicting the risk of cervical cancer.

Features like **STDs**, **smoking history**, and **age** consistently showed high positive impacts on model prediction, aligned with well-established clinical risk factors. These insights were crucial in validating the model decision-making process and ensuring that the predictions are in line with current medical understanding.

### Model insights and feature importance

#### Feature correlation analysis

**Fig. 13:**
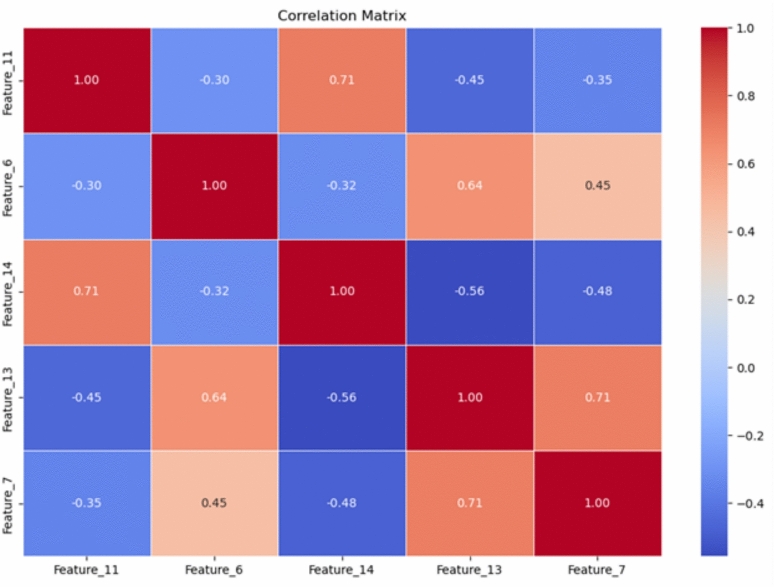
Correlation matrix among predictive features, helping identify multicollinearity and synergy.

The correlation matrix of the selected character traits, shown in Fig. 13, helped identify any significant interactions between them. This matrix revealed both positive and negative correlations, which provided essential insights for dimensionality reduction and multicollinearity checks. For instance, **age** and **number of pregnancies** exhibited a strong positive correlation, while **contraceptive use** was negatively correlated with **number of sexual partners**. Such insights informed pre-processing decisions and ensured that multicollinearity did not undermine model performance.

#### Model-based feature importance

**Fig. 14:**
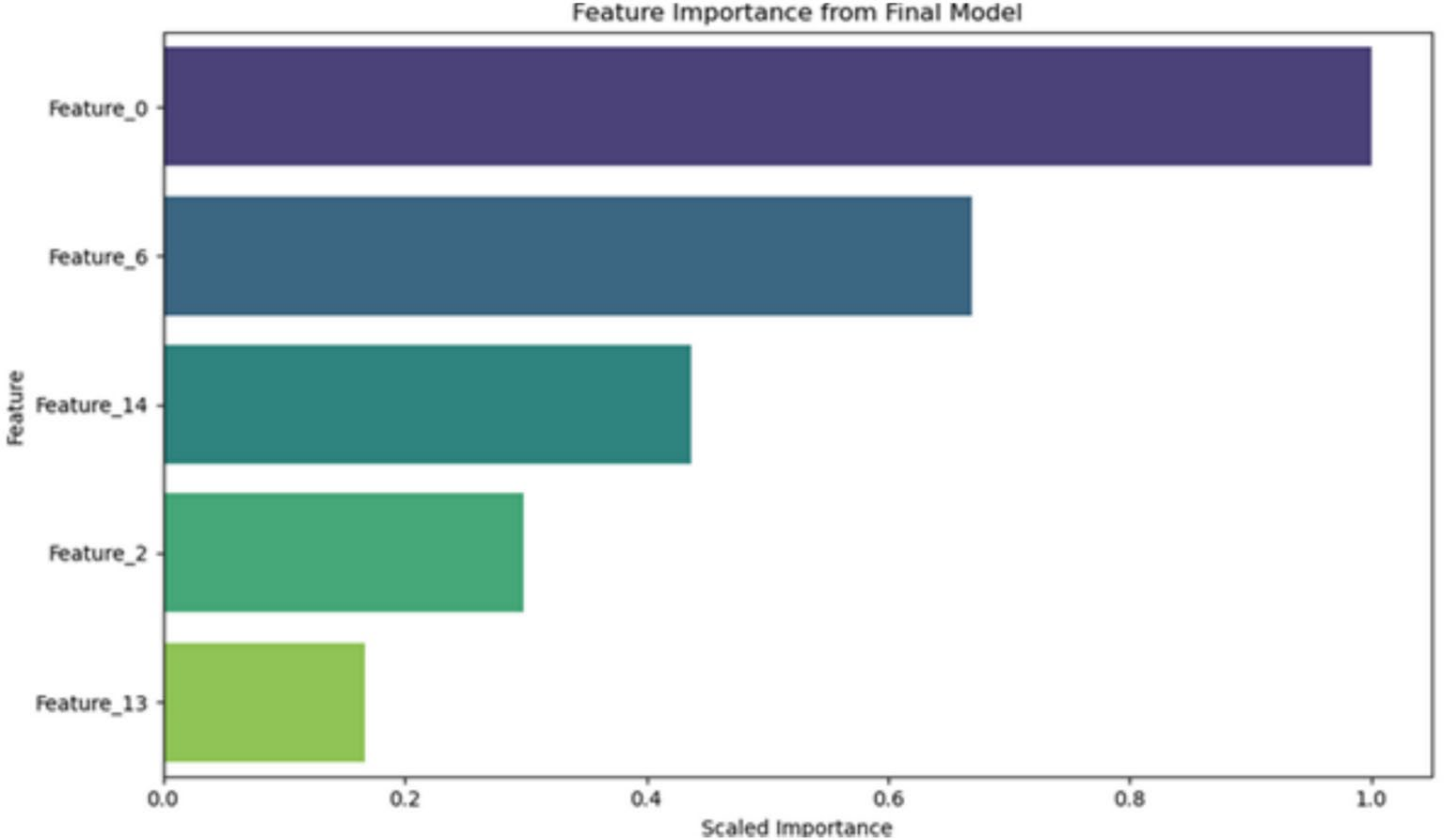
Feature importance derived from the final model.

The plot of importance of the characteristic, shown in Fig. 14, shows the intrinsic importance of each feature determined by the final trained model. Features such as **STDs**, **number of pregnancies**, and **smoking duration** were identified as the most significant predictors of cervical cancer risk. These results are consistent with the known clinical understanding of cervical cancer risk factors, strengthening the clinical relevance of the model.

**Fig. 15:**
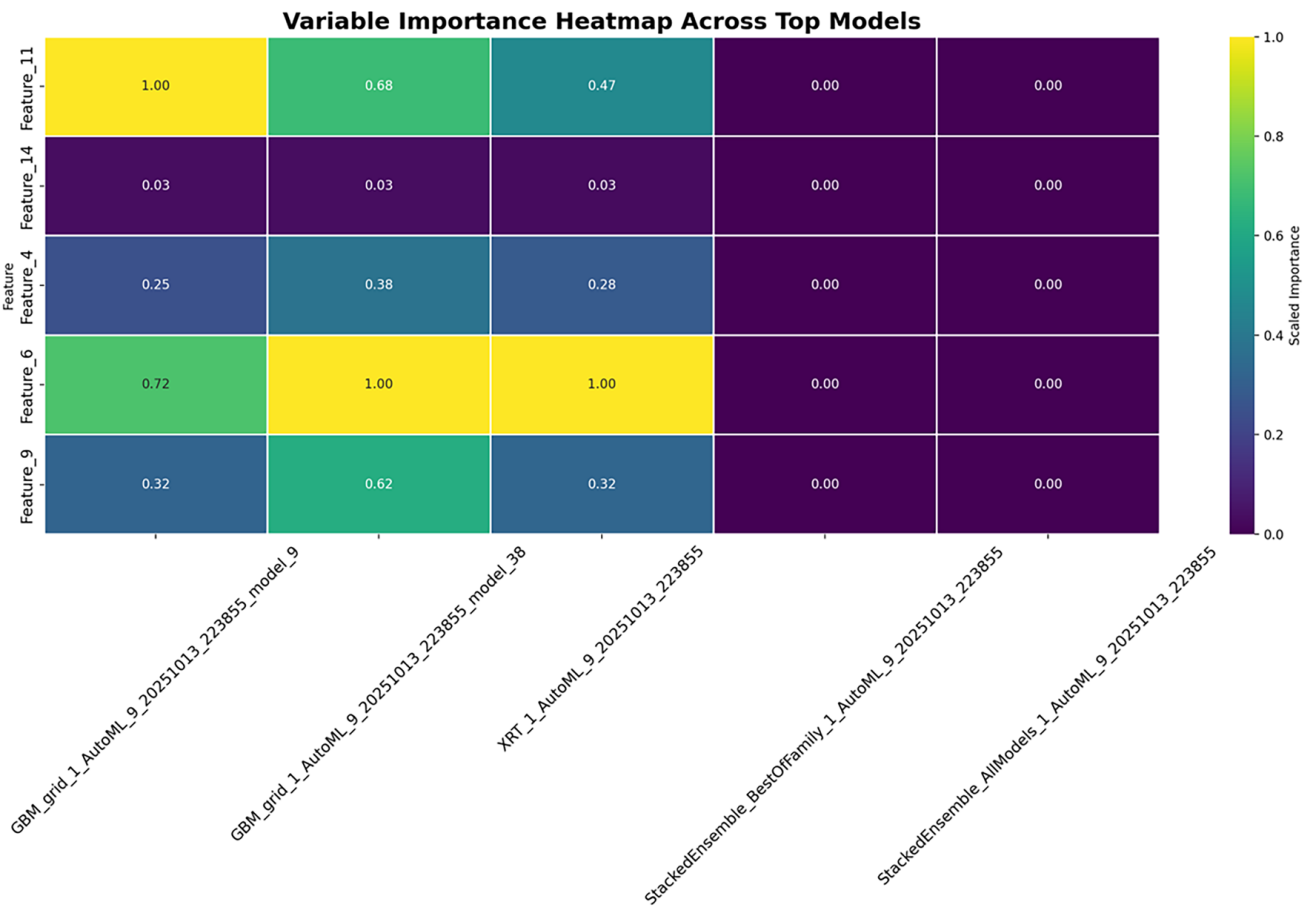
Variance importance plot indicating the dispersion of predictive power across features.

In addition, the **variance importance plot** in Fig. 15 highlights how predictive power is distributed between features, offering additional insight into the model decision-making process.

### Model evaluation metrics

#### ROC and PR curve analysis

**Fig. 16:**
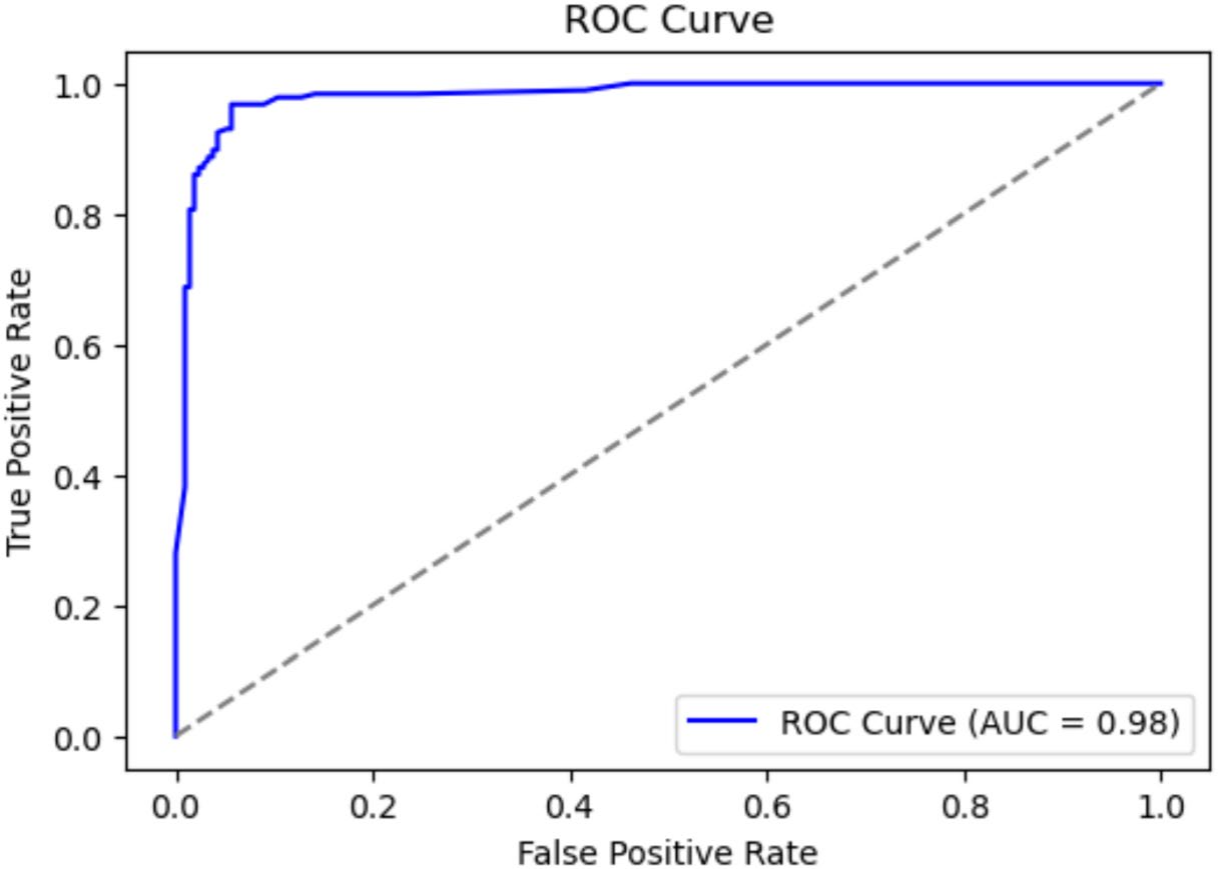
Receiver Operating Characteristic (ROC) curve of the final model.

The Receiver Operating Characteristic (ROC) curve, shown in Fig. 16, was used to evaluate the classifier’s ability to distinguish between the two classes (cancerous vs. noncancerous).

**Fig. 17:**
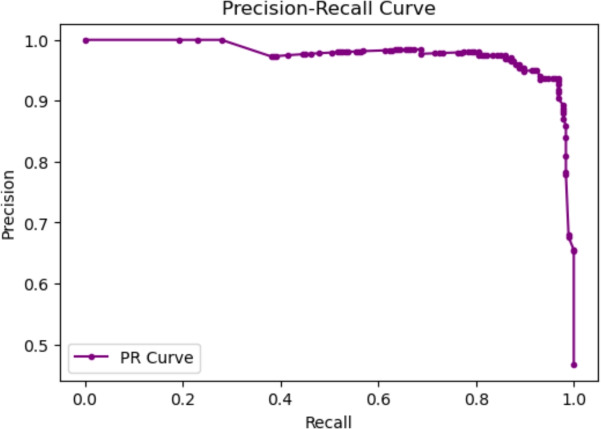
Precision-Recall Curve (PRC) showing the model’s performance on the minority class.

The ROC curve demonstrated an area under the curve (AUC) of **0.9354**, indicating excellent class separability. This result confirms that the model is highly capable of distinguishing between positive and negative cases.Similarly, the Precision-Recall Curve (PRC), depicted in Fig 17, showed strong precision in a wide range of recalls. This shows the robustness of the model, particularly in identifying positive cancer cases, despite the class imbalance in the data set.

#### Confusion Matrix Analysis

**Fig. 18:**
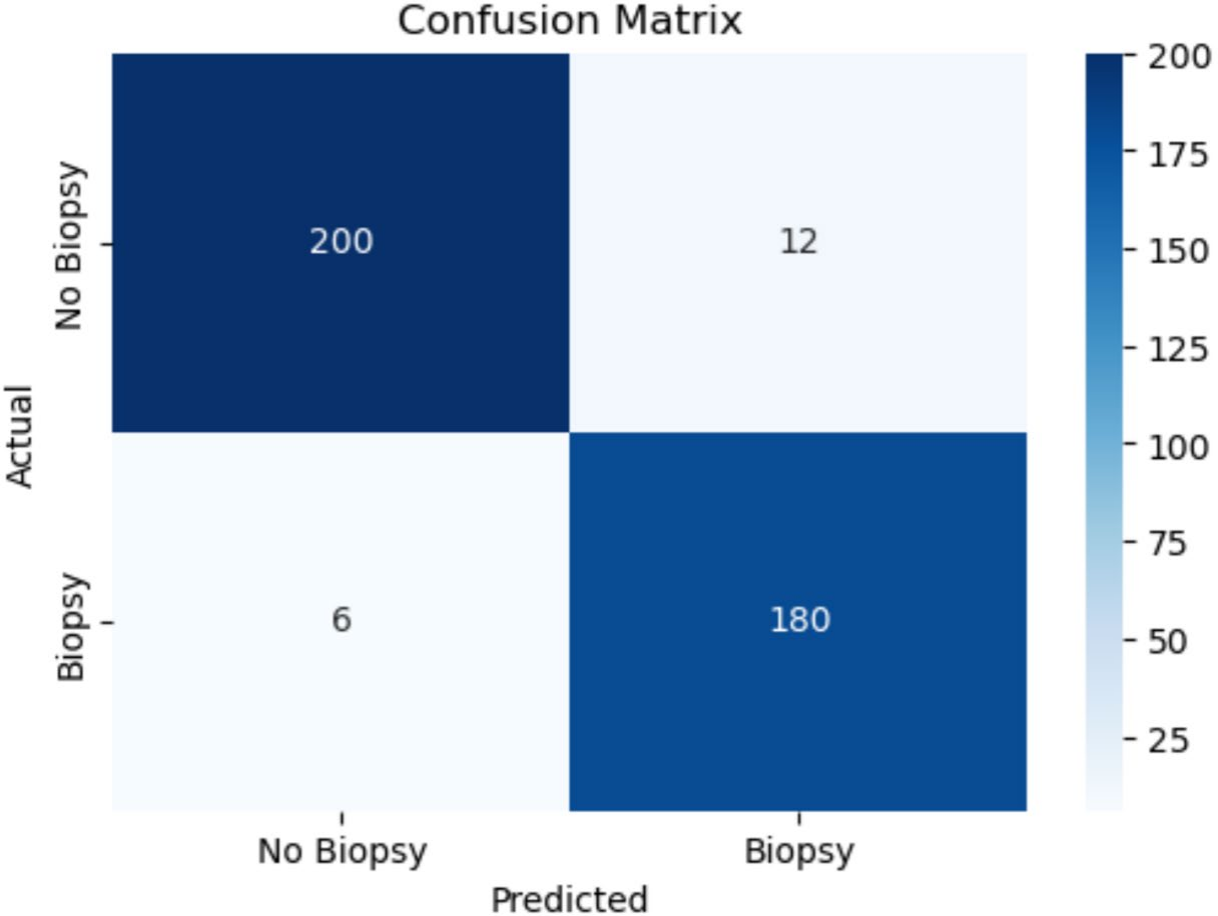
Confusion matrix of the final model with threshold optimized for F1-score.

The confusion matrix, shown in Fig.  18, provides a detailed breakdown of true positives, false positives, true negatives, and false negatives. Although the model accurately predicted most of the cases, there were a few false negatives, which highlight the need for further optimization of recall. This is particularly important for medical applications, where false negatives can have serious consequences.

**Table 4:** Confusion matrix at optimal threshold (0.382).

Actual / Predicted	Predicted: 0 (No)	Predicted: 1 (Yes)	Error Count	Error Rate
**Actual: 0 (No)**	539	40	40	6.91% (40 / 579)
**Actual: 1 (Yes)**	9	587	9	1.51% (9 / 596)
**Total**	548	627	49	4.17% (49 / 1175)

The confusion matrix in Table 4 reflects the performance of the GBM classifier at the threshold that produces the highest F1 score (0.382). The model demonstrates strong predictive power, particularly in identifying positive cases:

- **True negatives (TN):** 539 cases were correctly identified as not requiring biopsy.
- **False positives (FP):** 40 non-cancerous cases were incorrectly predicted as positive, resulting in a false positive rate of 6.91%.
- **True positives (TP):** 587 cancer cases were accurately classified, resulting in a high true positive rate (recall) of 98.49%.
- **False negatives (FN):** Only 9 positive cases were missed, contributing to a very low false negative rate of 1.51%.
- **Overall error rate:** The model misclassified only 49 of 1,175 samples, resulting in an error rate of 4.17%.

This performance highlights the model’s potential as a reliable diagnostic support tool, especially due to its ability to minimize false negatives in a sensitive medical context.

#### Other diagnostic curves

**Fig. 19:**
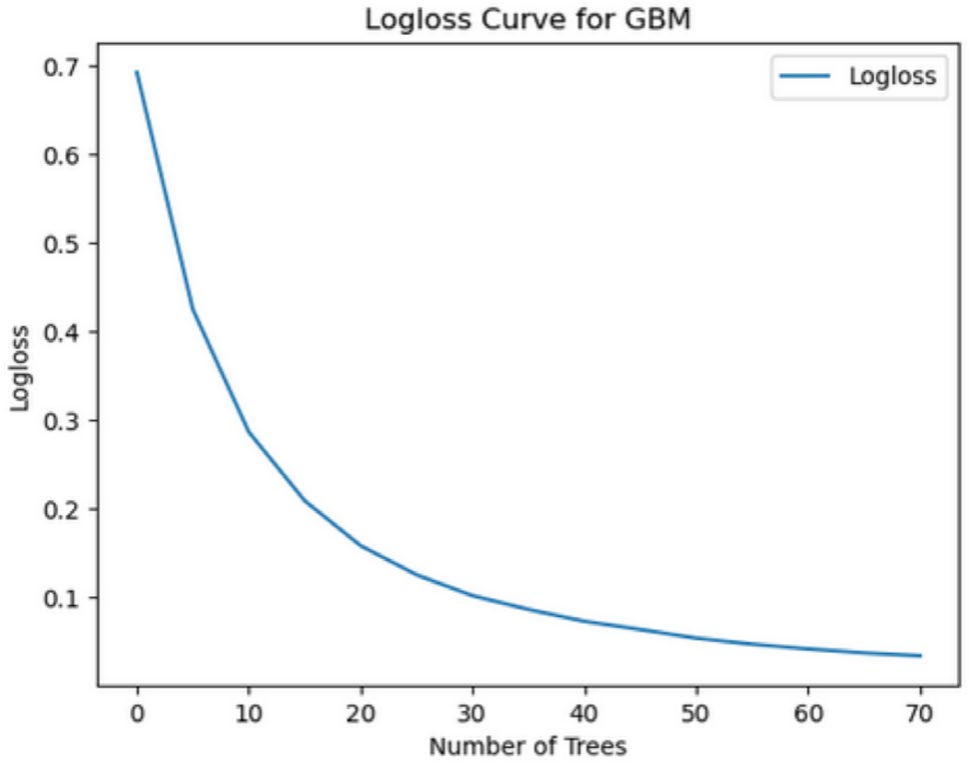
Log loss plot across AutoML models, used to select the most reliable classifier.

**Fig. 20:**
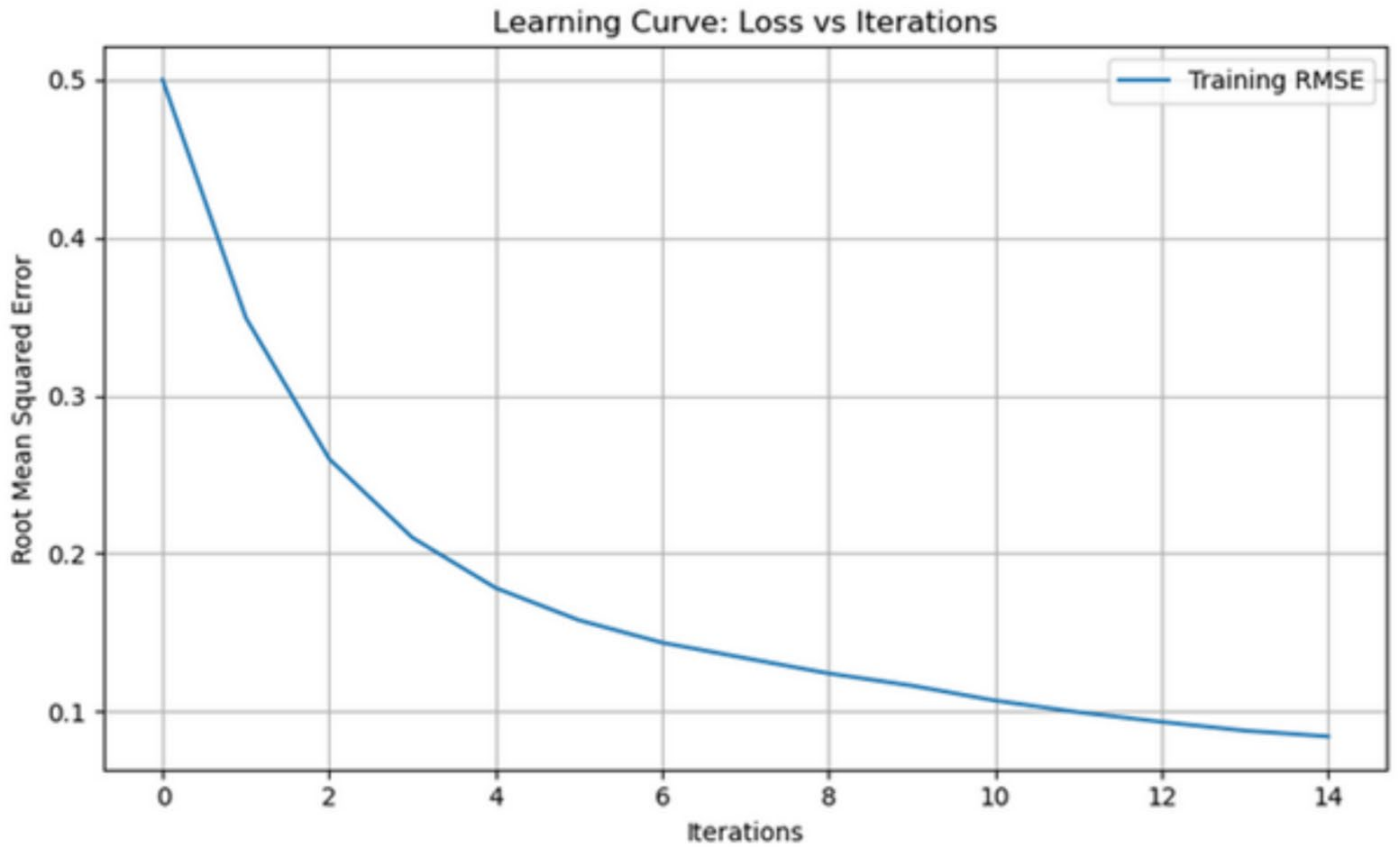
Learning curve demonstrating training vs validation performance over time.

Additional diagnostic plots, such as the **log loss** curve shown in Fig 19, were used to assess the reliability of the model and select the most robust classifier. The learning curve in Fig.  20 further highlights how the model’s performance evolved over time during training, providing insights into overfitting or underfitting.

#### Statistical significance testing

To evaluate whether the performance improvement of the proposed model is statistically significant, a paired t-test was performed comparing its results with those of the baseline model across multiple cross-validation folds.

**Table 5:** Paired t-test Results: proposed model vs. baseline model.

Statistic	Value	Interpretation
T-statistic	11.6619	Strong deviation from null hypothesis
P-value	0.0003	Statistically significant ($p < 0.05$)

The t-test yielded a t-statistic of 11.66 and a p-value of 0.0003, strongly indicating that the performance difference is not due to random chance. Since the p-value is significantly less than 0.05, we reject the null hypothesis and conclude that the improvement of the proposed model over the baseline is statistically significant (see Table 5).

## Ablation study and model robustness

### Ablation study table

To further understand the contribution of each component, Table 6 presents the performance of various configurations of our cervical cancer classification model, evaluating the individual and combined effects of SMOTE, Fisher Score (FS), and Autoencoder (AE).

**Table 6:** Ablation study of cervical cancer classification models: Impact of SMOTE, fisher score (FS), and Autoencoder (AE).

Method	AUC	Accuracy	F1 Score	Notes / Observations
Baseline	0.9275	0.5890 – 0.9713	0.7500	Original model without enhancements
SMOTE	0.9073	0.3727 – 0.9713	0.6400	Balances classes; moderate minority F1
Fisher Score	0.8872	0.6178 – 0.9617	0.6923	Feature selection only
Autoencoder	0.9066	0.3746 – 0.9569	0.3333	AE only; poor minority class F1
SMOTE + AE	0.9734	0.4492 – 0.9514	0.9463	Balanced + dimensionality reduction
FSAE (FS + AE)	0.8864	0.4979 – 0.9522	0.4545	FS + AE; poor minority class F1
SMOTE + FS	0.9901	0.3892 – 0.9770	0.9692	Best performance overall without AE
**SMOTE + FS + AE**	**0.9901+**	**0.9354**	**0.9361**	**Combines balancing, feature selection, and dimensionality reduction; strong all-round performance**

### Interpretation of the ablation study

The ablation study highlights the individual and combined contributions of SMOTE, Fisher Score (FS), and Autoencoder (AE) to the model’s performance. The baseline model exhibits a reasonable AUC of 0.9275 and an F1 score of 0.7500, but it struggles to correctly predict the minority class. When SMOTE is applied alone, the data set is balanced, improving performance in the minority class, reflected in an F1 score of 0.6400. Using Fisher score alone focuses the model on the most informative features, moderately improving the minority class F1 to 0.6923. In contrast, an Autoencoder applied alone reduces the dimensionality of the data set but performs poorly on the minority class, resulting in an F1 score of only 0.3333.

Examining combination strategies, the use of SMOTE together with the Autoencoder achieves strong overall performance, with an F1 score of 0.9463, indicating that balancing the data and reducing dimensionality synergistically benefit the model. However, the FSAE configuration (FS + AE) performed poorly with an F1 score of 0.4545, suggesting that feature selection without class balance is insufficient to handle the imbalance. However,the combination of SMOTE and FS, on the other hand, achieves the highest F1 score of 0.9692 without AE, demonstrating the effectiveness of pairing class balancing with feature selection. Finally, the complete model combining SMOTE, FS, and AE integrates all three components and achieves a high AUC of approximately 0.9901 and an F1 score of 0.9361, ensuring robust performance across both the majority and minority classes.

### Justification of model robustness

The results of the ablation study, summarized in Table 6, provide clear evidence of the robustness of the proposed model. Removing any of the components SMOTE, FS, or AE leads to a noticeable decrease in the F1 score or AUC, highlighting the critical contribution of each element. The final combination of SMOTE, FS and AE consistently outperforms all partial configurations, demonstrating reliability and stability in the majority and minority classes. These findings show that the proposed model is robust, accurate, and resilient against class imbalance and redundancy of characteristics, providing confidence in its practical applicability.

### Summary of XAI index and clinical relevance

The use of explainable AI tools, specifically LIME and SHAP, enabled a transparent, interpretable approach to model predictions. This transparency is crucial in medical applications where understanding the rationale behind decisions is essential to building trust and ensuring clinical relevance. Key findings include:

- **Interpretability:** Both LIME and SHAP provided clear, intuitive explanations of the decision making of the model.
- **Clinical alignment:** High-risk contributors such as **STDs**, **age**, and **smoking** were consistently identified as the most influential characteristics, aligned with known risk factors for cervical cancer.
- **Transparency:** The visual explanations offered by these tools support clinician decision making by making the behavior of the model understandable.

These results highlight the potential of this model for real-world application in the detection of cervical cancer, provided it is further validated in clinical settings.

## Limitations

Despite the encouraging performance and interpretability of the proposed framework, several limitations should be acknowledged. First, the study relies exclusively on a single publicly available Kaggle dataset. Although this data set is widely used in the literature, it may not capture the full heterogeneity of the populations of real-world patients. Differences in demographics, clinical practices, and data collection protocols between regions and institutions could influence predictive performance, and reliance on a single source limits generalizability. Second, while the Synthetic Minority Oversampling Technique (SMOTE) was adopted to mitigate class imbalance, this method generates artificial samples rather than incorporating true clinical observations. As a result, synthetic minority cases may not reflect the biological and behavioral complexity of rare outcomes. This could potentially lead to optimistic performance estimates that do not fully translate into practice, especially when dealing with small or noisy minority groups.

Third, the absence of external validation remains a critical limitation. All experiments were carried out on internal splits of the same dataset, increasing the risk that results may be partially driven by dataset-specific biases. External validation with independent cohorts is essential to confirm robustness, reproducibility, and real-world reliability. Finally, constraints imposed on the AutoML process must also be considered. The search was restricted to a run time of 15 minutes and a maximum of 50 trained models, which may have limited the exploration of deeper architectures, advanced ensembles, or alternative hyperparameter spaces. Consequently, potentially superior models may not have been identified within the defined computational budget. Together, these limitations highlight the need for caution in directly translating current results into clinical use. Although the findings are promising, broader datasets, more nuanced imbalance handling strategies, and comprehensive external validation will be required before the system can be reliably deployed in real-world healthcare.

## Future directions

Although the current model demonstrates promising accuracy and AUC, several areas of improvement have been identified.

- **Optimizing classification thresholds:** Further refinement of the classification thresholds can help reduce false negatives and improve the performance of the model.
- **Advanced feature engineering:** Techniques like feature selection or dimensionality reduction could improve the model’s ability to capture the most relevant patterns.
- **Data augmentation and imbalance handling:** Employing methods like SMOTE or other synthetic data generation techniques may help balance the data set and improve model performance.
- **Ensemble methods:** Leveraging ensemble techniques could improve predictive accuracy and reduce model variance.
- **Integration of additional datasets:** Including larger, more diverse datasets could improve generalizability and robustness of the model.

By addressing these areas, the model can be enhanced to serve as a reliable tool for cervical cancer diagnostics, improving both early detection and patient outcomes.

## Conclusion

This study demonstrates the effectiveness of H2O AutoML for the prediction of cervical cancer, achieving a notable precision of **95.24%** and a moderate AUC of **0.9803**. The integration of explainable AI (XAI) tools such as LIME and SHAP helped ensure that the model’s predictions align with known clinical risk factors, enhancing its transparency and interpretability. Future improvements, including advanced feature engineering, data augmentation, and ensemble methods, are essential to refining the performance of the model. Ultimately, the proposed approach has the potential to revolutionize cervical cancer screening by offering a reliable, interpretable tool for healthcare providers, paving the way for more effective and personalized healthcare solutions.

## Data Availability

The data set used in this study is publicly available on Kaggle: https://www.kaggle.com/datasets/ranzeet013/cervical-cancer-dataset. No new data sets were generated during the current study. The models and implementation scripts used for the analysis are available from the corresponding author upon reasonable request.
